# Abstract Animations for the Communication and Assessment of Pain in Adults: Cross-Sectional Feasibility Study

**DOI:** 10.2196/10056

**Published:** 2018-08-03

**Authors:** Charles R Jonassaint, Nema Rao, Alex Sciuto, Galen E Switzer, Laura De Castro, Gregory J Kato, Jude C Jonassaint, Zakia Hammal, Nirmish Shah, Ajay Wasan

**Affiliations:** ^1^ Center for Behavioral Health and Smart Technology Department of Medicine University of Pittsburgh Pittsburgh, PA United States; ^2^ Human-Computer Interaction Institute Carnegie Mellon University Pittsburgh, PA United States; ^3^ Department of Medicine University of Pittsburgh Pittsburgh, PA United States; ^4^ Department of Psychiatry University of Pittsburgh Pittsburgh, PA United States; ^5^ Department of Clinical and Translational Science University of Pittsburgh Pittsburgh, PA United States; ^6^ Center for Health Equity Research and Promotion Veterans Affairs Pittsburgh Healthcare System Pittsburgh, PA United States; ^7^ Clinical Protocol and Data Management University of Pittsburgh Medical Center Pittsburgh, PA United States; ^8^ The Robotics Institute Carnegie Mellon University Pittsburgh, PA United States; ^9^ Division of Hematology Department of Medicine Duke University Durham, NC United States; ^10^ Department of Anesthesiology University of Pittsburgh Pittsburgh, PA United States

**Keywords:** pain, pain measurement, chronic pain, medical informatics, mobile apps

## Abstract

**Background:**

Pain is the most common physical symptom requiring medical care, yet the current methods for assessing pain are sorely inadequate. Pain assessment tools can be either too simplistic or take too long to complete to be useful for point-of-care diagnosis and treatment.

**Objective:**

The aim was to develop and test Painimation, a novel tool that uses graphic visualizations and animations instead of words or numeric scales to assess pain quality, intensity, and course. This study examines the utility of abstract animations as a measure of pain.

**Methods:**

Painimation was evaluated in a chronic pain medicine clinic. Eligible patients were receiving treatment for pain and reported pain more days than not for at least 3 months. Using a tablet computer, participating patients completed the Painimation instrument, the McGill Pain Questionnaire (MPQ), and the PainDETECT questionnaire for neuropathic symptoms.

**Results:**

Participants (N=170) completed Painimation and indicated it was useful for describing their pain (mean 4.1, SE 0.1 out of 5 on a usefulness scale), and 130 of 162 participants (80.2%) agreed or strongly agreed that they would use Painimation to communicate with their providers. Animations selected corresponded with pain adjectives endorsed on the MPQ. Further, selection of the electrifying animation was associated with self-reported neuropathic pain (*r*=.16, *P*=.03), similar to the association between neuropathic pain and PainDETECT (*r*=.17, *P*=.03). Painimation was associated with PainDETECT (*r*=.35, *P*<.001).

**Conclusions:**

Using animations may be a faster and more patient-centered method for assessing pain and is not limited by age, literacy level, or language; however, more data are needed to assess the validity of this approach. To establish the validity of using abstract animations (“painimations”) for communicating and assessing pain, apps and other digital tools using painimations will need to be tested longitudinally across a larger pain population and also within specific, more homogenous pain conditions.

## Introduction

At least 116 million adults in the United States are affected by chronic pain [1]; that is, pain lasting for more than 3 months [2]. Medical treatment and lost productivity due to pain costs approximately US $635 billion each year, more than the cost of treating cardiovascular disease, cancer, or diabetes [3]. More than 80% of patients presenting for a physician visit report pain as a primary complaint, and a further 80% of these patients receive inadequate treatment for their pain [4]. Accurate assessment and diagnosis of pain is necessary to provide appropriate pain treatment and quality care [5,6].

Despite the development of many validated pain assessment scales, there has been little advancement in pain assessment since the introduction of the McGill Pain Questionnaire (MPQ) in 1970 [7] and the FACES pain scale for pediatric patients in the 1980s [8]. The traditional approach of self-report scales reduces the complexity of pain to a number or to unidimensional statements of pain [9]. Even the most recently developed pain scales or apps still rely on numerical scales and pain adjectives to assess pain, despite evidence that patients struggle with expressing pain to clinicians on static forms that ask them to estimate their pain on a 0 to 10 scale [10]. This oversimplification of pain ignores the sometimes intermittent nature and moment-to-moment variation in key features of the pain experience. Thus, medical providers miss opportunities to relate to their patients and may miss important symptoms and related diagnoses, leading them to intervene on poorly described and ill-defined targets [11,12].

In addition, current pain assessment approaches may perpetuate disparities in health care. Overly complex pain measures that rely on a long list of adjectives may alienate people with low literacy, those with disabilities, seniors with dementia, and many others with communication limitations [13-15]. Racial or ethnic differences in pain perception and expression may not be accurately captured by simplified pain scales [16]. Because the pain report is almost entirely subjective, even white race patients without language limitations can have their pain needs misinterpreted, their symptoms ignored, or their credibility challenged [15].

Advances in technology have made it possible to improve tools that measure patient-reported outcomes and, in turn, allow for a higher quality of data capture [17]. However, the pain assessment scales now being offered in electronic formats are essentially copies of the paper-pencil scales and do not capitalize on the flexibility of electronic media [18]. The introduction of novel, patient-centric pain assessment tools that leverage technology will maximize health care providers’ ability to diagnose and treat pain.

This study tested the feasibility and utility of an innovative pain assessment tool that uses graphical illustrations and abstract animations (“painimations”) to measure pain quality and intensity. We proposed that using animations to assess pain would overcome the barriers of traditional pain scales, allow patients to more accurately communicate the pain experience, and potentially facilitate pain diagnosis and treatment. Initial development and testing of this concept focused on neuropathic pain because of the well-defined characteristics that differentiate it from other pain types, the high prevalence of neuropathy in chronic pain populations [19], and the availability of validated neuropathic pain scales for comparison [20,21]. This paper describes the design process behind a novel, animation-based pain assessment app called Painimation, as well as the performance characteristics and capabilities of using this app to measure pain and to detect any neuropathic pain component.

## Methods

### Conceptualization and Development of Painimation

The initial goal of this instrument development effort was to improve our understanding of the patient pain experience and address limitations of current pain assessment and treatment. The first step in this approach was to interview patients who experienced acute and chronic pain. These interviews used principles of contextual inquiry, a common method in the development of interactive applications, to quickly uncover users’ perceived needs [22]. In the first set of interviews, 10 patients were asked to recount both successful and unsuccessful encounters with clinicians regarding their pain, using a directed storytelling approach, an ethnographic research method where the participant discusses their past experience, with probes from the interviewer to elicit more detail on the underlying motivations and breakdowns [23]. The most prominent message from both chronic and acute pain patients was that “pain is so hard to describe and explain” and the “exact feeling is impossible to communicate.” In the second part of the interviews, participants were given the Brief Pain Inventory [24] to complete in a “think-aloud” protocol [25] where they completed the questionnaire while stating aloud what they were thinking as they worked through each item. Both chronic and acute pain patients expressed confusion around the Brief Pain Inventory 0-10 scale and found the experience of using it “vague” and “ambiguous.” Participants were also confused because the concept of “worst possible pain” is different for each person. Participants said, “I have no clue what [10 out of 10 pain] actually means.” Additionally, participants said that the scale does not allow for the varied experience of pain; for instance, one might want to say, “It’s an 8 when applying pressure, 7 when resting, and 10 early in the morning;” they described this limitation as “a lack of specificity.”

A thematic analysis of these interviews indicated that a new pain assessment would need to both accommodate the vague, inexact feelings people often have regarding their pain and enable people to indicate different types of pain at the same time. Taking into account pain literature that suggests pain is more accurately depicted visually [26] and is better communicated with word pictures, analogies, and metaphors [27,28], we decided to use a highly visual, abstract, and expressive mode of pain communication: animations.

To develop the animations, the investigators started out with a list of words used to describe qualities of pain and reduced this list to several groups. We created animations to represent each group of sensations. Initial animations were tested with a group of 16 participants (see Rao [10] for more description). After some revision and another iteration of design, a final set of eight animations was created and selected for testing. To simplify the reporting of results and identification of animations, the animations were loosely labeled as “pounding,” “shooting,” “throbbing,” “tingling,” “cramping,” “burning,” “stabbing,” and “electrifying.”

We next developed an iOS app called Painimation that allowed users to select one or more of the eight animations previously listed. Development of Painimation involved an iterative process with three phases. In Phase 1, a group of six patients and family members were shown a demonstration of the initial version (v1.0) of the app and contributed input that was used to revise the app. In Phase 2, a group of five different participants who suffered from chronic pain were given a tablet device with the revised version of the Painimation app (v1.1) for testing. They were asked to enter their current pain symptoms and typical pain over the past 2 weeks. Data from this round of user testing was used to refine the app again. Finally, in Phase 3, all participants from Phase 2 were approached and asked to enter data using the close-to-final version of the Painimation app (v1.2) to confirm that all their concerns expressed in Phase 2 testing had been addressed. Any additional concerns raised during the Phase 3 testing were addressed in the finalized version of the app (v1.3).

### App Description

**Figure 1:**
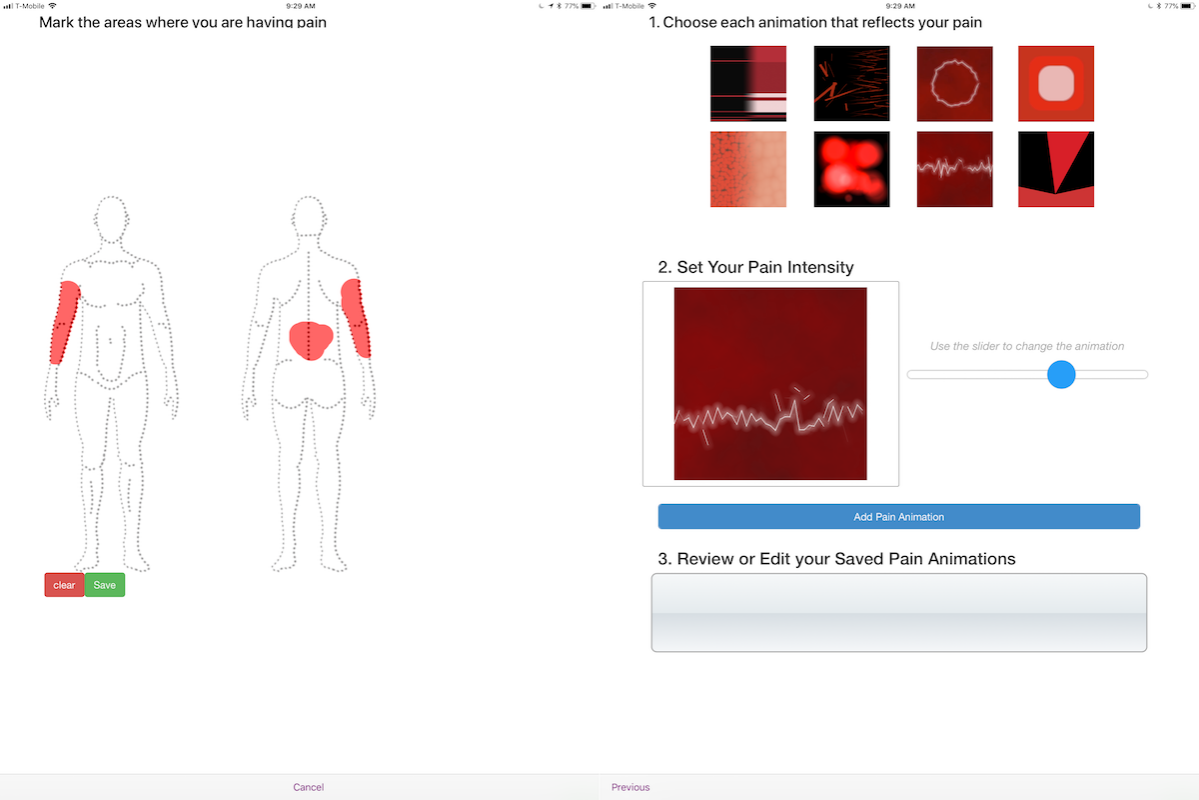
Screenshots of the Painimation app that uses images and animations to assess pain location, quality, and intensity.

Painimation first shows users a body image and asks them to mark the areas where they are having pain. If they make a mistake, they are able to clear their markings and start again. Once they are satisfied with the selection of pain locations, they save this image and advance to the next screen where they are shown a selection of eight animations, which they then use to describe the quality of their pain (Figure 1; Multimedia Appendix 1). Once the user selects an animation, he or she is asked to indicate the intensity of their pain by using a slider to change the animation “intensity.” Moving the slider changes the animation intensity by increasing or decreasing its speed, color saturation, focus, and size. The user can manipulate the animation until they feel it most closely matches or reflects the quality and intensity of their pain experience. The position of the intensity slider (where the lowest position is 0 and the highest position or maximum is 100) is used as the pain intensity score for that specific animation. When the user is satisfied with their selected and customized pain animation, they add the animation to a bin, at which point they can either select another animation or proceed to the next screen. The app allows up to five animations to be added to the bin. Participants can review each of their chosen animations at the final screen and are then presented with the patient satisfaction questionnaire. The backend of the app provides both the qualitative data (pain location and type) and quantitative data (pain intensity and percentage of body covered in pain) on pain symptoms.

### Proof-of-Concept Study Methods

#### Study Population

We tested Painimation in the general pain medicine clinic at the University of Pittsburgh Medical Center (Pittsburgh, PA) in adult patients (≥18 years of age) currently receiving treatment for chronic pain. In order to be eligible, patients had to have experienced pain more days than not for at least three consecutive months including the day of study participation. At clinic check-in, eligible patients were given a postcard by the clinic receptionist with a brief description of the study and instructions to approach one of the research assistants in the waiting room if interested. To maintain patient privacy, we did not obtain data on nonparticipants. Those patients who were interested received a tablet computer from a research assistant; the tablet guided the potential participant through the electronic consent process.

#### Electronic Consent Process and Data Capture

Using the Apple iOS ResearchKit framework facilitated the electronic consent process and completion of study questionnaires on an Apple iPad. Patients who elected to participate in the study and signed the electronic consent form were presented with a brief battery of electronic questionnaires that they were asked to complete while in the waiting room. All data, including a portable digital file of the signed consent, was uploaded directly from the ResearchKit app to a REDCap database [29].

### Measures

Participants first completed a basic demographics form and a brief clinical history of their pain, in which they self-reported whether or not they currently had pain, the severity of current pain on a visual analog scale (VAS), the duration of pain condition, and the type of pain condition, choosing from the following: nerve damage, arthritis, sickle cell, fibromyalgia, back pain, neck pain, headache, migraine, joint pain, chronic pain, abdominal pain, or other.

Participants completed the MPQ [7]; the PainDETECT [20] questionnaire, a measure of neuropathic pain; and the experimental Painimation app*.* Each participant was randomized automatically to complete the Painimation scale first or last. The randomization of questionnaires helped determine if seeing the pain adjectives on the MPQ and/or the pain statements on PainDETECT influenced selection of animations or satisfaction with the measure.

The MPQ consists primarily of three major classes of word descriptors—sensory, affective, and evaluative—that are used by patients to specify subjective pain experience. These word descriptors from the MPQ were correlated with each animation in the Painimation app. Mapping the words participants used to describe their pain to which animation they chose gave a sense of how participants may have been interpreting the animations.

PainDETECT is a screening tool for neuropathic pain, with seven weighted sensory descriptor items and two items relating to the spatial (radiating) and temporal characteristics of the individual pain pattern. Compared to clinical diagnosis, its sensitivity is 85% and its specificity 80%. PainDETECT was initially developed and validated in patients with back pain but also has shown applicability to patients with other types of neuropathic pain. For screening purposes, cut-off scores of 12 or less (a neuropathic component is unlikely) and 19 or greater (a neuropathic component is likely) are recommended [20].

Painimation is a novel app, developed by our group, for pain assessment using graphical illustrations and abstract animations to measure pain location, quality, and intensity. See a more detailed explanation of the app in the “App Description” section earlier in Methods. Data for Painimation were stored both on a backend server and on the iPad device. We confirmed data in both locations for consistency.

Patient satisfaction was evaluated once the user completed entering their pain on Painimation. Users were asked if it (1) was useful for describing their pain, (2) enjoyable to use, and (3) would be useful for communicating their pain to the provider; for each item, users were asked to choose a response on a 1 to 5 Likert scale from 1=strongly disagree to 5=strongly agree. We hypothesized that using the Painimation app would be associated with high patient satisfaction regarding usability of the app and ability to communicate their pain experience.

### Statistical Analysis

#### Distributions and Comparison by Demographics

Descriptive measures of central tendency and dispersion were used to examine distributions of pain scores. The three pain scale scores were evaluated for differences by age (above and below the median), sex, race/ethnicity, and location of pain to determine if there were differences in pain intensity and quality.

#### Discriminate Ability of Painimation

To examine how well Painimation could accurately discriminate between different pain types, the study compared patients’ animation selection to their pain diagnosis (eg, nerve damage, arthritis, or sickle cell disease), as well as differences in Painimation-recorded pain quality (ie, the painimation[s] they chose) by self-report pain type. The primary analysis for this study was the one-way comparison between neuropathic pain, self-report nerve damage, and nonneuropathic pain. We characterized the association between specific Painimation scores and pain diagnosis with means and Pearson correlations.

Next, to compare Painimation to more traditional pain measures (PainDETECT, the MPQ, and the VAS), we examined distributions of pain scores across all measures using descriptive measures of central tendency, and the associations between measures using Pearson correlation coefficients for continuous measures and phi correlation coefficients when comparing two dichotomous outcomes. The discriminate ability of Painimation versus PainDETECT measures was measured by first using chi-square analysis to compare the association of the PainDETECT-recommended cut-off score (scores ≤12 suggest neuropathic component is unlikely) with self-report nerve damage, versus selection of the electrifying animation with nerve damage.

To test sensitivity and specificity for detecting a neuropathic pain component for Painimation and PainDETECT we calculated receiver operating characteristic (ROC) curves derived using logistic regression analyses, quantified by area under the curve (AUC). To accomplish this for Painimation, the analysis took into account animation intensity (speed/saturation) and transformed the electrifying animation into a continuous 0 to 100 score by modeling nonselection of electrifying as “0” and, for those who selected “electrifying,” using the intensity value they selected for that animation. Using the continuous PainDETECT score and “electrifying” animation score (ie, participant-selected intensity of the animation), we tested confidence intervals of the two AUCs to determine whether the confidence intervals for AUC overlapped between the two measures. We performed linear regression with the response variable being the pain type and the type of measure (Painimation or PainDETECT) as the independent variable.

We also examined the correlation between the electrifying animation and specific qualitative descriptors on the PainDETECT scale by assessing the association of the animation with each questionnaire item. The PainDETECT questionnaire uses an 11-point ordinal scale for intensity of each qualitative descriptor. For each number, we calculated the Painimation intensity mean with 95% confidence intervals, and the median with interquartile ranges. We tested whether 1-point change in the PainDETECT questionnaire was associated with increased probability of a participant’s selecting the “electrifying” animation.

## Results

### Characteristics of Study Sample

The study obtained consent from 202 participants. We removed from analyses 13 participants who were missing data on more than one of the full questionnaires. The excluded sample did not differ from the other participants on any variables of interest. Reasons for missingness were random (ie, ran out of time, being called to clinic room for their physician visit, or poor Internet connection). The analyzed sample (N=189) had a mean age of 51.55 (SD 13.86) years, with 124 of 189 (65.6%) reporting female gender, 123 of 189 (65.1%) reporting white race, and 45 of 189 (23.8%) reporting black or African American race. Of the 189 participants, 66 (34.9%) participants had a college degree or higher, whereas only 12 (6.3%) had less than a high school/GED education. A majority of patients presented with back pain (123/189, 65.1%), chronic pain syndrome (108/189, 57.1%), arthritis (78/189, 41.3%), and nerve damage (67/189, 35.4%).

Due to data errors specific to the Painimation app, we lost study data for n=19 participants. These participants did not differ on any variables of interest from the n=170 participants with complete Painimation data. For the individuals with complete data (n=170), the animations most frequently selected were electrifying (66/170, 38.8%), throbbing (54/170, 31.8%), cramping (51/170, 30.0%), burning (51/170, 30.0%), shooting (47/170, 27.6%), and stabbing (35/170, 20.6%). The tingling (10/170, 5.9%) and pounding (21/170, 12.3%) animations were selected least frequently.

### Associations of Painimation Selections With Clinical Features

The animations selected by pain patients showed several associations with clinical features of the patients. Mean current pain level on the VAS was 6.8 (SE 0.2, range 0-10). Those who chose the “electrifying” animation had a significantly higher VAS pain level (mean 7.2, SE 0.2) than those who did not (mean 6.3, SE 0.3; *P*=.02); those who chose the “stabbing” animation had a marginally higher VAS pain level (mean 7.2, SE 0.2) than those who did not (mean 6.6, SE 0.2; *P*=.15), but this was not statistically significant. There were no differences on the VAS pain scale for any other animations.

Several animations were associated with different types of pain (Table 1). Neuropathic pain was associated with the “electrifying” animation and marginally associated with the “shooting” animation. Fibromyalgia was associated with the “pounding” and “tingling” animations. Headache-type pain was associated with the “pounding” and “tingling” animations and marginally with the “electricity” animation. Abdominal pain was associated with the “pounding” animation. There were no other notable associations. The PainDETECT score was associated with all pain types except abdominal pain and “other” pain types. PainDETECT score showed the strongest correlation with the “electrifying” and “shooting” animations, and was also correlated to a lesser extent with the “pounding” and “stabbing” animations.

Tables 2 and 3 show associations between pain diagnosis, the “electrifying” animation, and the dichotomized PainDETECT score. The “electrifying” animation was associated with nerve damage and marginally associated with general headache. The dichotomized PainDETECT score was not associated with nerve damage, but was associated with fibromyalgia, back pain, neck pain, headache pain, and chronic pain syndrome.

Each painimation that was associated with specific MPQ pain quality descriptors by at least 10 participants is presented in Table 4. The “electrifying” painimation was associated with the MPQ descriptors sharp, burning, and tingling. The “pounding” painimation was associated with stabbing, cramping, and sore. “Shooting” was associated with pulsing and sharp; “cramping” with stabbing; “throbbing” with pulsing, sore, and hurting; “tingling” with throbbing; “burning” with cramping and sore; and “stabbing” with pulsing, stabbing, and burning. PainDETECT score was correlated with each of the top selected MPQ descriptors except throbbing, sore, hurting, and aching.

**Table 1:** Correlation matrix showing associations between self-report pain diagnosis, Painimation, and PainDETECT total score (n=170).

Self-report diagnosis	Expressive Painimation animations, *r*								PainDETECT score, *r*
	Electrifying	Pounding	Shooting	Cramping	Throbbing	Tingling	Burning	Stabbing	
Nerve damage	.159^a^	.017	.141	.019	–.010	.021	.046	.135	.165^a^
Arthritis	.084	.045	–.017	–.008	–.040	–.009	–.008	–.077	.149^a^
Fibromyalgia	.054	.170^a^	–.013	.026	.133	.188^a^	.026	.109	.283^c^
Back pain	.083	–.059	–.011	.027	–.051	–.025	–.027	.072	.208^b^
Neck pain	–.047	–.012	.083	–.036	–.033	.000	.104	–.016	.293^c^
Headache	.138	.154^a^	.093	.067	.016	.212^b^	–.067	.070	.274^c^
Migraine	.120	.067	.069	.078	–.007	.086	.010	.001	.249^c^
Joint pain	.069	.049	.017	–.052	–.006	.045	.026	.047	.192^a^
Chronic pain	.112	.100	.150	.033	–.012	.009	.086	.048	.336^c^
Abdominal pain	–.054	.206^b^	–.133	.051	.058	.093	.051	–.049	.079
Other	–.055	.068	.036	.015	.038	.127	.130	–.066	–.025
PainDETECT score	.353^c^	.160^a^	.259^c^	.025	.004	.015	.007	.187^a^	—

^a^*P*<.05.

^b^*P*<.01.

^c^*P*<.001.

**Table 2:** Association between pain diagnosis and selection of the “electrifying” animation among adults with chronic pain (n=170).

Diagnosis	Selected electrifying animation, n (%)			*P* value
	No	Yes	Total	
Nerve damage	31 (29.8)	30 (45.5)	61 (35.9)	.04
Arthritis	40 (38.5)	31 (47.0)	71 (41.8)	.27
Sickle cell	4 (3.8)	1 (1.5)	5 (2.9)	.38
Fibromyalgia	19 (18.3)	15 (22.7)	34 (20.0)	.49
Back pain	64 (61.5)	46 (69.7)	110 (64.7)	.28
Neck pain	33 (31.7)	18 (27.3)	51 (30.0)	.54
General headache	14 (13.5)	16 (24.2)	30 (17.6)	.07
Migraine headache	14 (13.5)	15 (22.7)	29 (17.1)	.12
Joint pain	40 (38.5)	30 (45.5)	70 (41.2)	.37
Chronic pain syndrome	56 (53.8)	43 (65.2)	99 (58.2)	.15
Abdominal pain	27 (26.0)	14 (21.2)	41 (24.1)	.48
Other	15 (14.4)	7 (10.6)	22 (12.9)	.47

**Table 3:** Association between pain diagnosis and PainDETECT score among adults with chronic pain (N=186).

Diagnosis	PainDETECT score^a^, n (%)				*P* value
	Low	High	Total		
Nerve damage	28 (31.1)	39 (39.4)	67 (35.4)	.23	
Arthritis	33 (36.7)	45 (45.5)	78 (41.3)	.22	
Sickle cell	1 (1.1)	4 (4.0)	5 (2.6)	.21	
Fibromyalgia	7 (7.8)	29 (29.3)	36 (19.0)	<.01	
Back pain	51 (56.7)	72 (72.7)	123 (65.1)	.02	
Neck pain	17 (18.9)	40 (40.4)	57 (30.2)	.01	
General headache	7 (7.8)	26 (26.3)	33 (17.5)	.01	
Migraine headache	8 (8.9)	25 (25.3)	33 (17.5)	.01	
Joint pain	30 (33.3)	46 (46.5)	76 (40.2)	.07	
Chronic pain syndrome	36 (40.0)	72 (72.7)	108 (57.1)	<.01	
Abdominal pain	19 (21.1)	26 (26.3)	45 (23.8)	.41	
Other	12 (13.3)	13 (13.1)	25 (13.2)	.97	

^a^PainDETECT scores can range from 0 to 38. Scores of 0-12 suggest nociceptive pain or a neuropathic pain component is unlikely (<15% likelihood), scores of 13-18 suggest an unclear or ambiguous pain type, and scores of 19-38 suggests neuropathic pain component (>90% likelihood).

**Table 4:** Correlation matrix showing associations between McGill Pain Questionnaire (MPQ) descriptors, Painimation, and PainDETECT total score (n=170).

MPQ	Expressive Painimation animations, *r*									PainDETECT score, *r*
	Electrifying	Pounding	Shooting	Cramping	Throbbing	Tingling	Burning	Stabbing		
Pulsing	.063	.105	–.182^a^	.098	.236^b^	–.009	–.005	.166^a^	.237^b^	
Throbbing	–.079	.035	.036	.040	.149	.163^a^	–.001	.097	.052	
Pounding	–.041	.025	.001	.068	.015	.108	.022	.052	.197^b^	
Shooting	.083	.124	.109	.023	.033	–.017	.021	.116	.288^c^	
Stabbing	.066	.168^a^	.086	.208^b^	–.076	.064	–.061	.224^b^	.208^b^	
Sharp	.216^b^	.097	.250^b^	.068	–.064	.040	–.020	.087	.275^c^	
Cramping	–.107	.185^a^	–.012	–.044	.135	.070	.173^a^	.021	.189^a^	
Burning	.254^c^	.007	.047	.146	–.020	–.022	.044	.169^a^	.375^c^	
Tingling	.204^b^	–.006	.074	.042	–.100	.024	–.004	.144	.269^c^	
Sore	.073	.162^a^	–.039	.063	.170^a^	.031	.153^a^	.075	.138	
Hurting	–.002	.037	–.002	.009	.162^a^	–.052	–.065	.102	.082	
Aching	.134	.065	.073	–.069	.071	–.032	.034	.086	.110	

^a^*P*<.05.

^b^*P*<.01.

^c^*P*<.001.

### Sensitivity and Specificity of Measures to Predict Neuropathic Pain Component

We performed a ROC analysis to determine the ability of Painimation to discriminate a neuropathic pain component in this population, then compared to PainDETECT, a measure already validated for identifying neuropathic pain. In this proof-of-concept study, Painimation simply used selection of the “electrifying” animation and the selected intensity (0-100) as the correlate for neuropathic pain component. The AUC, relating to the sensitivity and specificity, was low for both PainDETECT (AUC=0.60, 95% CI 0.52-0.69) and Painimation (AUC=0.59, 95% CI 0.51-0.67). The comparison of the AUC for the two measures showed no significant difference in their ability to detect nerve damage in this population (χ^2^_1_=0.01, *P*=.90).

### Evaluation of Patient Satisfaction

At completion, 141 of 162 participants (87.0%) agreed or strongly agreed that Painimation was useful for describing their pain, 137 of 162 (84.6%) agreed or strongly agreed that Painimation was enjoyable to use, and 130 of 162 (80.2%) agreed or strongly agreed they would use Painimation to communicate their pain to providers.

## Discussion

### Data Support Use of Animations For Communicating Pain

This study explored the use of abstract animations and graphical illustrations as a novel method for assessing pain quality, intensity, and location in adult patients with chronic pain. Although preliminary, these data suggest that a technology-based approach that allows patients to express their pain experience using animations that they can adjust and customize is acceptable, and potentially more efficient than traditional methods of pain assessment.

We believe using animations to measure pain can not only allow patients to describe pain sensations in a similar manner to how they experience them but, by not relying on words or numeric scales, can remove language and literacy as potential barriers to pain assessment. Further, given the well-documented gender, ethnic, and cultural differences in the experience and expression of pain [16,30], a more nuanced measure that eliminates linguistic and cultural biases may help highlight and elucidate group differences. Painimation has the potential to benefit both researchers and clinicians: for the former, it can improve understanding of gender and ethnic differences in pain and, for the latter, it can decrease the frequency of misunderstandings in pain reporting. To our knowledge, there are currently no other pain assessment tools that address pain location, intensity, and quality in a short assessment format that is not limited by language or literacy. Both the FACES scale and VAS, although short in length, are too simplistic and fail to identify the location and quality of pain. Longer multidimensional pain assessment measures such as the MPQ and PainDETECT are burdensome to complete and are not appropriate for all literacy levels. Other mobile device apps, such as Catch My Pain and My Pain Diary, have pain location, pain intensity, and symptoms tracking; however, these pain apps are not able to capture pain type or quality, and lack diagnostic potential.

Indeed, the use of abstract animations to measure pain is a truly novel approach that has not been previously described. Although significant associations in the expected direction were identified, these associations were not very strong, which may suggest patients were interpreting these animations in very different ways, potentially due to the diversity of pain types included in the sample. More data will be needed to understand how patients interpret the animations and the subsequent implications for pain assessment methodology.

### Preliminary Evidence of Diagnostic Properties

Testing of Painimation showed that its “electrifying” and “shooting” pain animations were associated with patient-reported nerve damage. These associations were similar to the PainDETECT score with respect to neuropathic pain (*r*=.165). Further, there was no significant difference in specificity and sensitivity of the two measures in predicting nerve damage. The PainDETECT cut-off score was not associated with patient-reported neuropathic pain, whereas the “electrifying” animation was. This suggests that Painimation performed just as well as PainDETECT as a marker of neuropathic pain and has the added benefits of being a much shorter, more engaging, and less complex assessment tool. In fact, using Painimation to detect nerve damage required consideration of only one item, that being the selection or nonselection of “electrifying” pain, and ignored other data inputs such as location, intensity, and other animations selected. The AUC for both measures was modest, but these data provide a foundation for iterative development of Painimation and analytic approaches that may enhance the diagnostic properties of this assessment tool.

### Clinical Implications

The American Pain Society guidelines recommend that a numeric pain intensity rating (0-10) be recorded at every clinical encounter. However, this widespread effort to assess pain with a unidimensional numeric scale did not improve the quality of pain treatment for patients [31]. In fact, this initiative may have contributed to the rise in opioid prescriptions [32]. The 0 to 10 scale has not improved pain assessment or treatment because the scale is unidimensional, uninformative, and lacks utility for both patients and their medical care providers [10]. Technologies such as Painimation can be easily implemented into the clinical setting and provide as much or more information than traditional multidimensional pain assessment tools. Such technology could help reestablish pain as the fifth vital sign, helping to realize the original American Pain Society vision for routine pain assessment in clinical care.

Painimation has the potential to allow patients to express their pain in a way not previously possible and improve their communication with providers. One older patient with shingles said about using Painimation, “This is the first time I’ve been able to describe my pain to someone.” Painimation could also help reduce the time it takes for a comprehensive pain assessment and diagnosis. Current pain assessments rely on long interviews or questionnaires that burden patients and can impede clinic flow. One report found that hospitalized participants with cancer took approximately 24 minutes to complete the paper version of the MPQ [33] (range 12-45 minutes). Painimation may provide the same level of data on pain in a shorter time frame. Unfortunately, the current study was unable to collect exact time-to-completion data for each questionnaire due to having administered the questionnaires in a clinic waiting room setting where unpredictable interruptions at times invalidated timestamp data. However, the available data suggests that participants completed Painimation, on average, in less than 2 minutes. Future studies will administer this set of questionnaires to a subset of the sample in an isolated room to determine more accurate and valid time-to-completion data.

### Study Limitations

Despite the interesting nature of the data, our study has several limitations. First, pain type was assessed via self-report, which may not be as accurate as a medical diagnosis. In future studies, to more accurately and definitively identify a neuropathic pain component, it would be helpful to include objective measures of nerve damage such as electromyogram and nerve conduction studies. However, these measures are imperfect given that electromyogram typically does not detect small fiber neuropathies [34], and nerve conductions studies can have false positive results [35]. Second, this study was cross-sectional and the stability of measurements was not assessed over time. Third, although we assessed participant satisfaction with Painimation, we did not collect this data for the other pain scales; therefore, we could not compare participants’ satisfaction with Painimation to their satisfaction with the other pain scales. Finally, the pain population was mixed and may not have been the most appropriate for the first test of Painimation given that each pain disorder may be associated with a very unique pain experience. Our primary comparison measure, PainDETECT, also did not perform well in this population, which provides more evidence of population heterogeneity but also exposes the limitations of existing measures. Despite these shortcomings, however, study data show patterns in the expected direction and suggest that using animations to communicate and assess pain is feasible even in a mixed-pain population. Future studies of this technology may benefit from tailoring animations to specific pain populations.

In the current version of the Painimation app, the user identifies pain location(s) and then selects animations that reflect the quality and intensity of their pain. If multiple pain locations are selected, the app does not allow the user to identify which pain location the animation they selected references. The ability to apply animations to a specific body location will be a feature available on future versions of the Painimation app, but was not a feature of the app version tested in this study. In addition, a significant portion of study data was lost due to app errors. App complications were in part due to the challenge of delivering eight animations simultaneously. The study team worked to correct and prevent technical errors; however, future studies will benefit from rigorously testing the robustness of app functions and data transfer before full launch of the study.

### Future Research Directions

This report provides an initial evaluation of the utility of animations and graphical illustrations to describe pain quality, type, and severity. Future studies will further evaluate both qualitative and quantitative data regarding patients’ perceptions of Painimation and usability compared with other pain assessment tools. It will be important to increase the specificity and sensitivity of the tool by tailoring and testing it with specific pain types (eg, low back pain), in acute versus chronic pain, and with pain in specific diseases (eg, pancreatitis or sickle cell disease). Future longitudinal assessments will be able to test whether Painimation scores predict clinical outcomes such as likelihood of response to pain treatment and risk of rehospitalization. In addition, we will be testing a mobile-phone version of Painimation that allows patients to assess and report their pain daily.

### Conclusions

We have set out to change the pain assessment conversation and forge a new direction for research on patient-reported outcomes. This research contributes a patient-centric, automated, pain assessment method that has the potential to not only improve patient-provider communication, but to also generate higher quality patient-reported pain symptoms data to guide diagnoses and treatment. By using animations to assess pain, we can decrease the burden of long, detailed pain assessments while collecting pertinent information on each patient’s pain experience through an easy-to-administer, novel, and engaging medium. Further research with animation-based pain assessment tools is needed to improve this tool and to more definitively determine its validity and utility.

## Appendix

Multimedia Appendix 1 The attached mp4 file is uploaded to illustrate two of the painimations from the app.

## Abbreviations

AUC: area under the curve
MPQ: McGill Pain Questionnaire
ROC: receiver operating characteristic
VAS: visual analog scale
